# Multidisciplinary Tumor Board Smart Virtual Assistant in Locally Advanced Cervical Cancer: A Proof of Concept

**DOI:** 10.3389/fonc.2021.797454

**Published:** 2022-01-03

**Authors:** Gabriella Macchia, Gabriella Ferrandina, Stefano Patarnello, Rosa Autorino, Carlotta Masciocchi, Vincenzo Pisapia, Cristina Calvani, Chiara Iacomini, Alfredo Cesario, Luca Boldrini, Benedetta Gui, Vittoria Rufini, Maria Antonietta Gambacorta, Giovanni Scambia, Vincenzo Valentini

**Affiliations:** ^1^ Radiation Oncology Unit, Gemelli Molise Hospital - Università Cattolica del Sacro Cuore, Campobasso, Italy; ^2^ Department of Woman, Child and Public Health, Fondazione Policlinico Universitario A. Gemelli Istituto di Ricovero e Cura a Carattere Scientifico (IRCCS), Rome, Italy; ^3^ Department of Woman, Child and Public Health, Catholic University of the Sacred Heart, Rome, Italy; ^4^ Fondazione Policlinico Universitario A. Gemelli Istituto di Ricovero e Cura a Carattere Scientifico (IRCCS), Rome, Italy; ^5^ Radioterapia Oncologica, Dipartimento di Diagnostica per Immagini, Radioterapia Oncologica ed Ematologia, Fondazione Policlinico Universitario A. Gemelli, Istituto di Ricovero e Cura a Carattere Scientifico (IRCCS), Rome, Italy; ^6^ Scientific Directorate, Fondazione Policlinico Universitario A. Gemelli Istituto di Ricovero e Cura a Carattere Scientifico (IRCCS), Rome, Italy; ^7^ Dipartimento di Diagnostica per Immagini, Radioterapia Oncologica ed Ematologia, Fondazione Policlinico Universitario Agostino Gemelli, Istituto di Ricovero e Cura a Carattere Scientifico (IRCCS), Rome, Italy

**Keywords:** locally advanced cervical cancer, multidisciplinary tumor board smart virtual assistant, artificial intelligence, virtual medicine support, chemoradiation (CRT)

## Abstract

**Aim:**

The first prototype of the “Multidisciplinary Tumor Board Smart Virtual Assistant” is presented, aimed to (i) Automated classification of clinical stage starting from different free-text diagnostic reports; (ii) Resolution of inconsistencies by identifying controversial cases drawing the clinician’s attention to particular cases worthy for multi-disciplinary discussion; (iii) Support environment for education and knowledge transfer to junior staff; (iv) Integrated data-driven decision making and standardized language and interpretation.

**Patients and Method:**

Data from patients affected by Locally Advanced Cervical Cancer (LACC), FIGO stage IB2-IVa, treated between 2015 and 2018 were extracted. Magnetic Resonance (MR), Gynecologic examination under general anesthesia (EAU), and Positron Emission Tomography–Computed Tomography (PET-CT) performed at the time of diagnosis were the items from the Electronic Health Records (eHRs) considered for analysis. An automated extraction of eHR that capture the patient’s data before the diagnosis and then, through Natural Language Processing (NLP), analysis and categorization of all data to transform source information into structured data has been performed.

**Results:**

In the first round, the system has been used to retrieve all the eHR for the 96 patients with LACC. The system has been able to classify all patients belonging to the training set and - through the NLP procedures - the clinical features were analyzed and classified for each patient. A second important result was the setup of a predictive model to evaluate the patient’s staging (accuracy of 94%). Lastly, we created a user-oriented operational tool targeting the MTB who are confronted with the challenge of large volumes of patients to be diagnosed in the most accurate way.

**Conclusion:**

This is the first proof of concept concerning the possibility of creating a smart virtual assistant for the MTB. A significant benefit could come from the integration of these automated methods in the collaborative, crucial decision stages.

## Introduction

Biological, radiological and clinical knowledge in the locally advanced cervical cancer (LACC) setting, as in all other fields of oncology, is growing on exponentially. Oncologists deal every day with many patients characterized by complex and heterogeneous phenotypes. The simultaneous elaboration of complex information is difficult even for experienced physicians and a significant amount of relevant information could be lost in the clinical decision process, as a direct consequence of the “information overload” (1).

The huge amount of data created in hospitals and populating complex data-lakes, stays largely unexploited and, in most of the circumstances, not organized at all. These general considerations make it clear that Artificial Intelligence (AI), a general term which covers the use of a computer algorithms to model intelligent processes (2, 3), is a field with potentially limitless applications in medicine and, more specifically, in oncology. Through Machine Learning methods, AI enables managing large amounts of data and allows smart data clustering for decision support in several knowledge areas.

The automated extraction and classification of actionable information from unstructured data (reports) represents a prerequisite for expanding “predictive” abilities and effectively tailoring patient treatments. Once unstructured and structured information are integrated and made consistent, and predictive methods are introduced to support diagnostic and therapeutic decisions, the most appropriate body where these data-driven methods can be exploited is the Multidisciplinary Tumor Board (MTB).

MTBs working groups have the main purpose in selecting the most appropriate and effective treatment for cancer patients, by taking into account staging of the tumor and its classification along with overall clinical characteristics. Several specialists often take part to the multidisciplinary meeting, such as radiation and medical oncologists, pathologists, radiologists, surgeons, nuclear medicine physicians and research nurses. Therefore, the point of views may be various and sometimes conflicting. Moreover, the discussion of each clinical case is often long and complex, especially if there are conflicting exams or if only the reports and not the images are available. Finally, there are not many cases that can be clearly discussed in a single MTB session.

AI and Machine Learning have already been used as a decision support tools in the framework of MTBs (4, 5) - yet many unmet needs are still voiced by MTB operators that may be addressed through such innovative approaches. The opportunities for more effective decision-making process can be summarized as follows:

- decision-making support by integrating different sources and information (as well as knowing which source is most reliable).
- decision-support systems that allow automated discrimination of simple vs. complex cases to help focusing efforts for the latter.
- reduce potential inconsistencies and lack of homogeneous criteria for diagnostic assessments by developing data-driven methods and common languages.
- enable increased teamwork and effective decision making across clinical expertise.
- leverage retrospective analyses from large data set to create methods and knowledge base that can be exported to other hospitals, thus creating a standardized approach for scalable methods and multicentric research efforts.

In our constant efforts to ameliorate the outcomes in the treatment of LACC, starting from the extensive work performed on chemo-radiation followed by surgery (5–10), we plan to implement a tailored AI-based decision support process. We blue-printed and implemented an automated system based on Natural Language Processing (NLP) (11, 12) to extract clinically relevant information from different free text reports of diagnostic exams and procedures that are commonly used in daily clinical activity, followed by a machine learning predictive method to support diagnostic decisions.

Therefore, to further develop and test the robustness of our automated system, we have performed a proof of concept by designing the first prototype of the “MTB Virtual Assistant” with the following goals:

- Automated classification of clinical stage starting from different free-text diagnostic reports;
- Resolution of inconsistencies by identifying controversial cases drawing the clinician’s attention to particular cases worthy for thorough multi-disciplinary discussion;
- Support environment for education and knowledge transfer to junior staff;
- Integrated data-driven decision making and standardized language and interpretation.

## Materials and Methods

### Patients

Data from patients affected by LACC, FIGO stage IB2-IVa, treated between 2015 and 2018 were extracted from our institutional data-lake. The following Electronic Health Records (eHRs) items have been considered for analysis:

- Staging Magnetic Resonance (MR) report;
- Gynecologic examination under general anesthesia (EUA) report;
- Staging Positron Emission Tomography–Computed Tomography (PET-CT) report.

Other patient’s relevant data (e.g., demographics, laboratory tests, body mass index, drugs, comorbidities etc.) were collected for further analysis.

### Methods

A two steps model has been applied to allow the set-up of the MTB Virtual Assistant:

- Automated extraction of the relevant eHR sets that capture the patient’s data before the diagnosis and then, through Natural Language Processing (NLP), analysis and categorization of all information to transform source information into structured data,
- development of A.I. methods to support the clinical staff in the decision process with regards to tumor staging confirmation and to help in identifying the most complex cases, where more complex analyses and discussion are needed (e. g. due to conflicting information coming from different exams).

A first subset of patients with pre-validated staging and diagnosis was used as training set for steps one and two.

Once steps (i) and (ii) have been completed and successfully tested for patients’ subsets with pre-validated staging and diagnosis (the ‘training set’), we developed an integrated toolset to support the MTB diagnostic process. Each time a new patient is selected for staging and treatment decision-making and enters the workflow, her eHR are automatically processed to provide structured clinical features (e.g. presence/absence of specific disease features in the tumor region, tumor activity etc.).

The A.I. algorithm then delivers an assessment for the staging of the tumor with a certain degree of reliability, reported on the screen as percentage of accuracy. The MTB staff can proceed– if needed- to go deeper in the characterization of the information, performing further analyses of clinical data patterns from different sources and comparing the content from different eHRs. This process, characterized by such a depth and complexity of information, and the A.I. empowered multi-dimensional analyses allow a robust consensus on the clinical decision to be taken.

### Step (i): Natural Language Processing: Extracting Clinical Data from Text-Based Medical Reports

The first step is represented by the extraction of clinically relevant information from MR, EUA, PET-CT reports and other eHRs. The challenge with these data sources was firstly to transform the unstructured information into discrete, categorical data able to define a clear, robust and actionable framework of clinical and pathological features related to the tumor loco-regional morphology.

The output of this transformation is therefore a pattern of structured clinical features that describe in detail the disease of the patient whose specific data constitute the source information of the integrated A.I. empowered analysis.

In terms of computer algorithm used, the NPL method to transform text into data is based on a hybrid approach using rules and annotations derived from medical guidelines, combined with A.I. (machine learning); in this experience, this was developed using the SAS Visual Text Analytics^®^ environment (12, 13). Pre-processing steps as such as segmentation, boundary detection and tokenization, and word normalization (stemming, spelling correction, expansion of abbreviation) were performed to achieve a higher degree of accuracy. Thereafter, syntactic and semantic analysis were performed with the support of an algorithm that creates the network of words, showing the occurrence of links among two words and providing an enhanced approach to natural language understanding. Finally, the sequence of steps above gave us the relevant NLP features leading to data extraction from real life medical reports.

By using these NLP steps, the medical reports were processed and free-text diagnostic information were transformed into categorical or quantitative clinical data that classify the clinical features resulting from each of the three exams MR, EUA, PET-CT. The selection of the relevant clinical features that characterize the diagnosis – and most importantly tumor staging – was performed by the multidisciplinary clinical team and constitute the basis for the ontology of the study.

Therefore, the result of this data discovery process for each patient is a table showing how detailed clinical features in the tumor region are diagnosed for each of the three exams – as shown in **Table 1A**. Any clinical feature is then inspected and reported as being or not within the framework of the three types of exams. Categorical morphological variables (i. e. whether or not a specific region is involved) are mostly extracted from MR and EUA, while PET-CT clinical features provide additional levels of tumor (metabolic) activity.

**Table 1A:** Clinical features included in the three diagnostic exams and data types.

	Data Type	Clinical Feature Included in diagnosis	Clinical Feature Included in diagnosis	Clinical Feature Included in diagnosis
		MR	EUA	PET-CT
Parametrium involvement	*Categorical*	o	o	
Vaginal lower third involvement	*Categorical*	o	o	
Vaginal middle third involvement	*Categorical*	o	o	
Vaginal upper third involvement	*Categorical*	o	o	
Bladder involvement	*Categorical*	o	o	
Rectum involvement	*Categorical*	o	o	
Vesico-vaginal septum involvement	*Categorical*	o	o	
Recto-vaginal septum involvement	*Categorical*	o	o	
Hydronephrosis	*Categorical*	o		
Lymph nodes involvement	*Categorical*	o		
Lymph nodes activity	*Quantitative*			o
Cervical lesion	*Categorical*	o	o	
Cervical activity	*Quantitative*			o
Fornix involvement	*Categorical*	o	o	
Stroma involvement	*Categorical*	o	o	
Methabolic activity	*Quantitative*			o
“Other” activity	*Quantitative*			o

MR: Magnetic resonance; EUA: Examination under anesthesia; PET-CT, Positron Emission Tomography–Computed Tomography.

Therefore, after the eHR automated reading and the subsequent NLP step, the patient’s clinical features are collected in a summarized pattern, as shown in **Table 1B** (specific instance of the table for a patient case); this view shows, for each of the clinical features, whether this has been identified as positive (meaning whether that region is involved in the tumor progression) or not. Examples from **Table 1B** indicate bladder involvement, as detected both by MR and EUA, while rectovaginal septum appears as involved when analyzing the results from the EUA and not from the RM. This conflicting outcome may indicate uncertainty in the staging assessment, which is typically represented in the predictive model results, as explained in step (ii) below.

**Table 1B:** Example of a patient’s pattern with convergent and conflicting features.

	Data Type	Clinical Feature Inspected (Y/N)	Clinical Feature Inspected (Y/N)	Clinical Feature Inspected (Y/N)
		MR	EUA	PET-CT
Parametrium involvement	*Categorical*	Y	Y	
Vaginal lower third involvement	*Categorical*	N	N	
Vaginal middle third involvement	*Categorical*	N	N	
Vaginal upper third involvement	*Categorical*	N	N	
Bladder involvement	*Categorical*	N	N	
Rectum involvement	*Categorical*	N	N	
Vesico-vaginal septum involvement	*Categorical*	N	N	
Recto-vaginal septum involvement	*Categorical*	Y	N	
Hydronephrosis	*Categorical*	N		
Lymph nodes involvement	*Categorical*	Y		
Lymph nodes activity	*Quantitative*			Y
Cervical lesion	*Categorical*	Y		
Cervical activity	*Quantitative*			Y
Fornix involvement	*Categorical*		Y	
Stroma involvement	*Categorical*	Y		
Methabolic activity	*Quantitative*			N
“Other” activity	*Quantitative*			N

MR: Magnetic resonance; EUA: Examination under anesthesia; PET-CT, Positron Emission Tomography–Computed Tomography; Y, yes; N, no.

This transformation from unstructured to structured data is the mainstay of the input to the prediction and clustering then executed by A.I. (machine learning) models.

### Step (ii): Assessment of Tumor Staging through Statistical Learning

**Figure 1:**
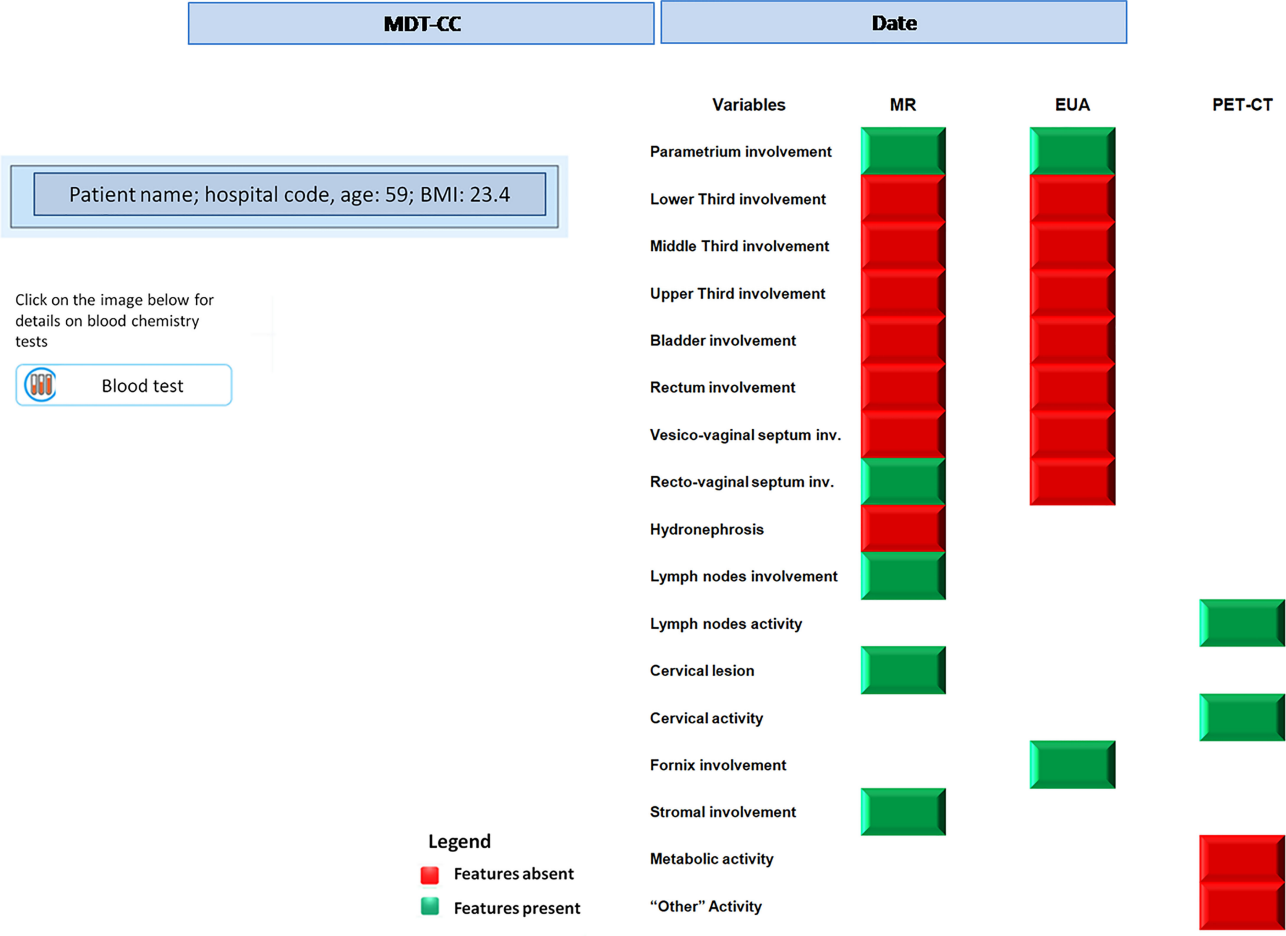
Example of dashboard showing clinical features from three diagnoses.

To create a system that supports the MTB in disease staging, the first step is to use a supervised learning technique for the training set, where tumor stage was known *a priori* for each patient in this group. This was achieved by applying clustering methods to classify patients based on similarity in their clinical feature pattern (the summary view as in **Figure 1**) and in their diagnosed staging. When applying clustering algorithms for each of the 3 diagnostic methods separately (MR, EUA, PET-CT) seven groups for each of the three diagnoses were generated, with a good degree of discrimination. Once the clusters have been created in the training set, a machine learning algorithm has then been used to build a predictive model for the staging based on composition of the clusters. “Decision Tree” algorithms have been adopted, using the SAS Vyia ^®^ analytics and modeling features.

Finally, a validation step has been performed on a new set of patients to predict their staging based on the trained Decision Tree model, testing the validity of the model.

## Results

The system has been firstly used to retrieve, with an automated extraction procedure, all the eHR for 96 patients with histological proven LACC. This represented and has been used as the training set of the study, with validated 2009 FIGO staging classification ranging from IB2 to IVA as output.

The available eHR included MR, EUA, and PET-CT diagnostic reports for all these patients.

The system resulted to be able to classify all patients belonging to the training set and - through NLP procedures - the clinical features were analyzed and classified for each patient. This analysis provided the patient-specific summary dashboard shown in **Figure 1** (desktop MTB team dashboard, which corresponds to **Table 1B**). This highlights how the different diagnostic methods have identified which areas have been impacted by the tumor progression (i. e. presence/absence of the disease in different regions) and the main activity levels. Again, this ‘clinical feature pattern’ also highlights when two different diagnostic methods have provided different outcomes for a given area, which is critical to identify patients who require a more thorough analysis during the MTB meetings.

**Figure 2:**
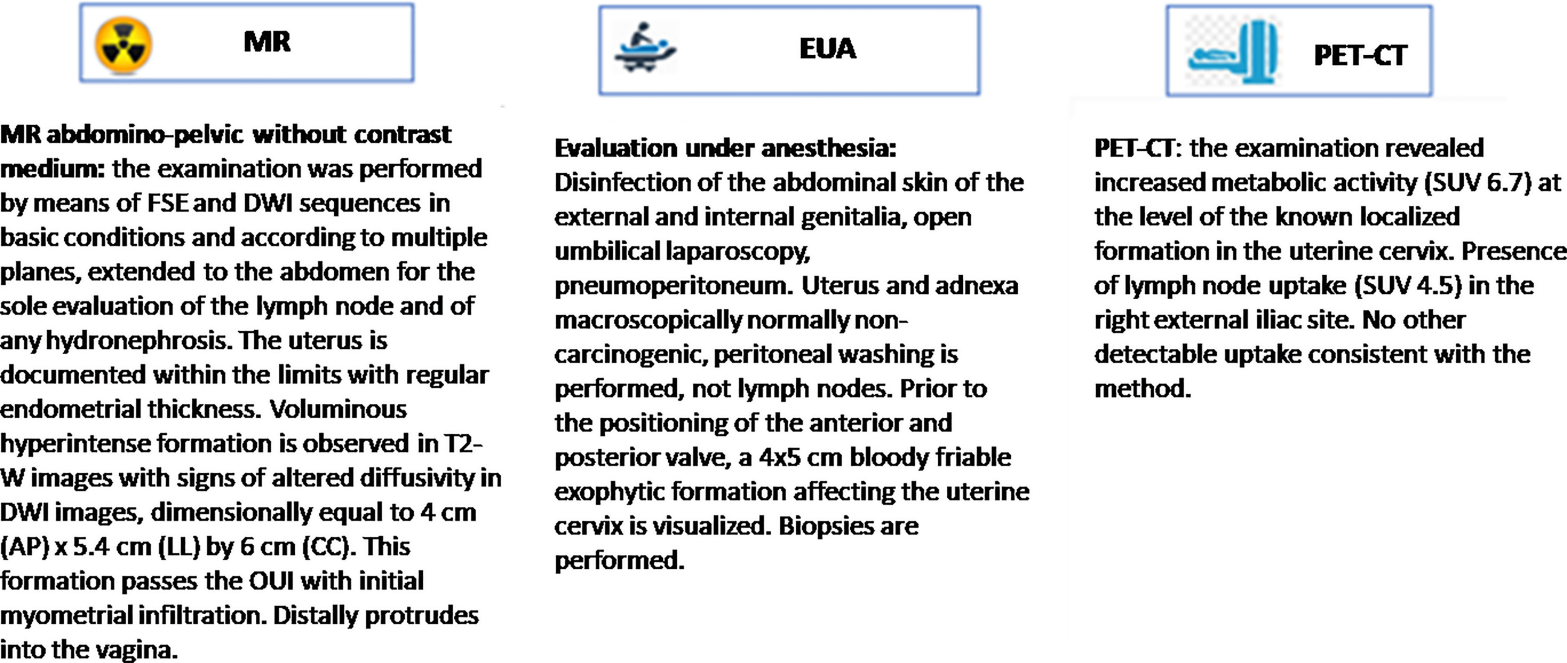
Example of dashboard included in Virtual Assistant that compare medical reports.

In addition, the clinical staff can retrieve other clinical parameters of interest directly from the system, such as laboratory exams, biomarkers, risk factors – and it is always possible to get the direct access to medical reports and compare them as shown in **Figure 2**.

Next, we focused on the development of predictive models for the 2009 FIGO staging classification based on the 96-patients worth training set and using a set of Decision Tree machine learning algorithms, obtaining a patient’s staging prediction accuracy of 94%.

The model uses clinical features extracted and classified from the MR and the EUA reports. Even higher accuracy (98%) can be achieved integrating the input from the PET-CT.

**Figure 3:**
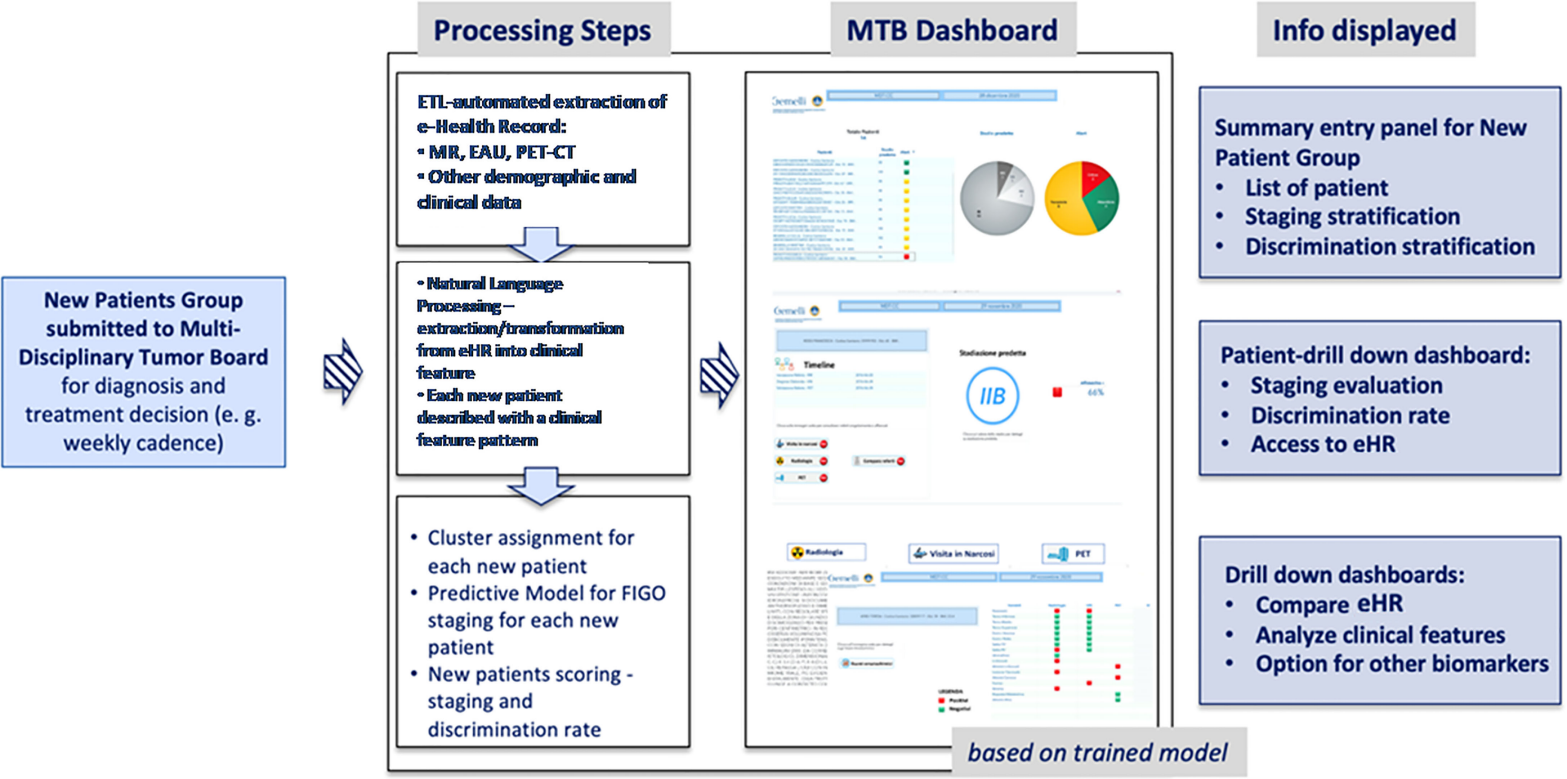
Logical view of the Virtual Assistant dashboard and use in Multidisciplinary Tumor Board.

However, we consider the staging prediction coming from MR and EUA combined as a more solid base for predictive methods, as these two exams evaluate the same morphological and anatomical indicators. In addition, they represent a consistent and replicable set of diagnosis that can be exported to other medical centers quite easily. Once the information dashboard and predictive model have been designed based on the training set, we have put focus in creating a user-oriented operational tool targeting the MTB and the clinical teams who are confronted with the challenge of large volumes of patients to be diagnosed in the most accurate way. The resulting decision support system is summarized in **Figure 3** in a logical diagram.

**Figure 4:**
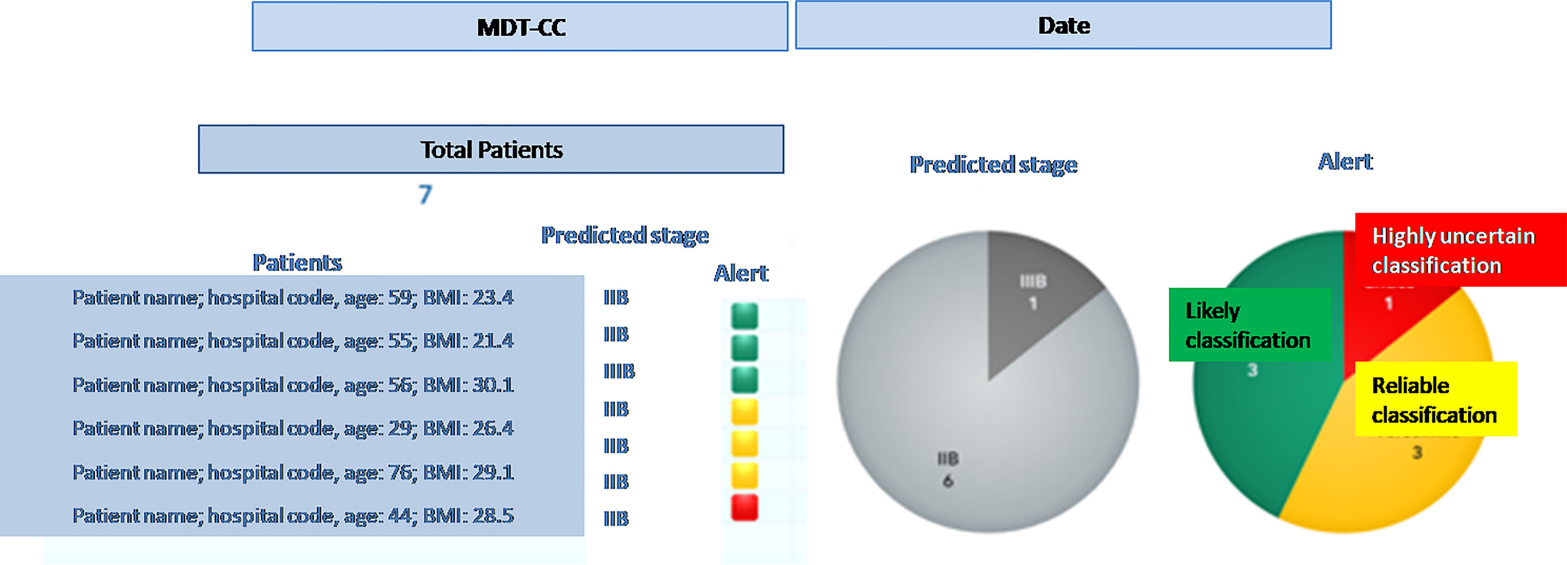
Entry dashboard that classify incoming patients for the Multidisciplinary Tumor Board session.

**Figure 5:**
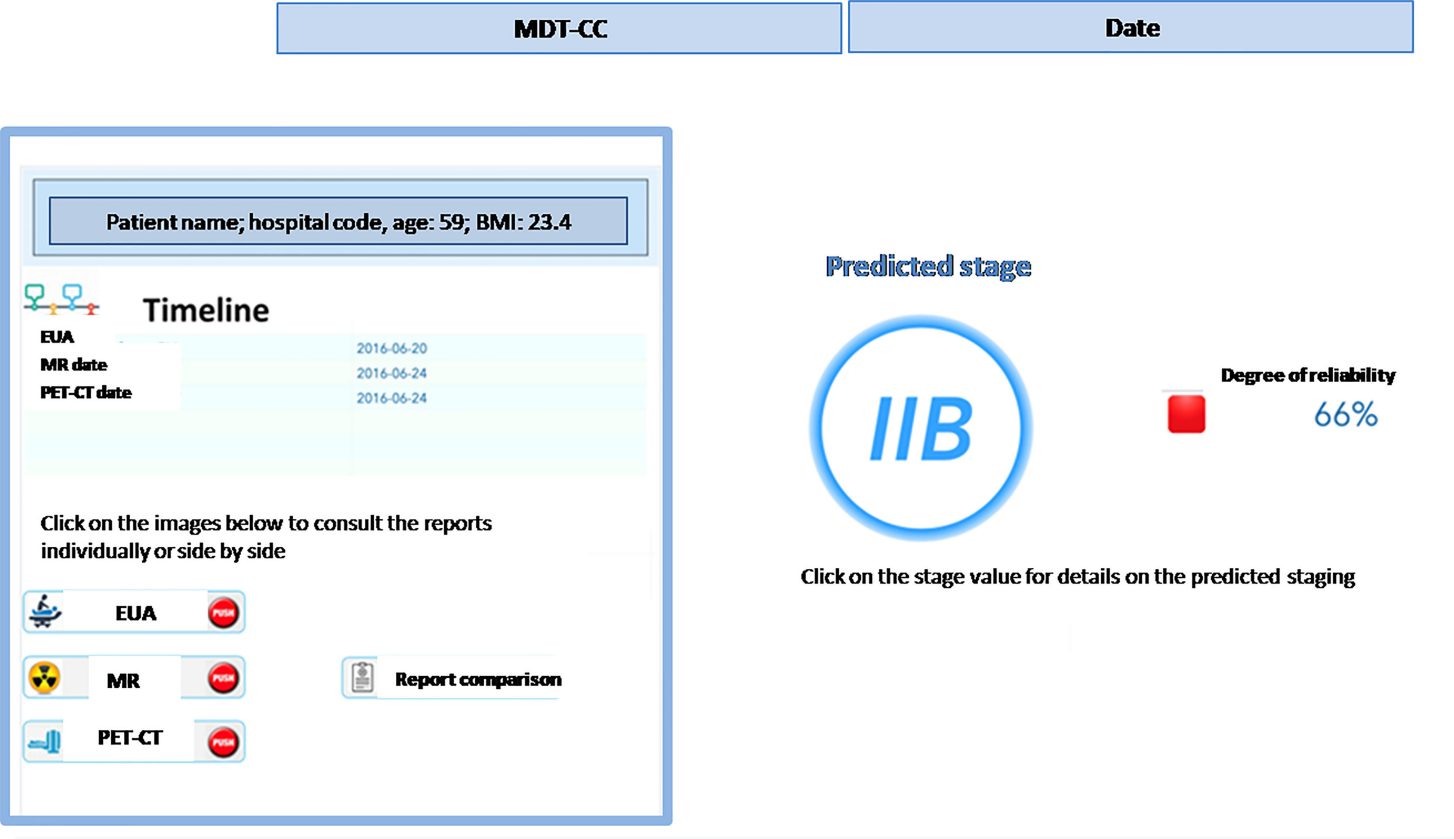
Dashboard view of a patient for the Multidisciplinary Tumor Board.

The flow to support the MTB is designed as follows:

- when a new patient is prepared for the discussion at the MTB, the system performs the following processing steps: (i) automatic retrieval of eHR and other clinical data; (ii) NLP based transformation of the free text reports into structured clinical features that characterize the single patient, on the basis of the three diagnostic exams (MR; EUA; PET-CT); (iii) clustering of patients according to the clinical features patterns; (iv) machine-learning based prediction of the pre-diagnostic FIGO staging;
- once the steps described above have been completed (in near-real time) for any new patient, the MTB staff will be able to consult the list of patients covered in the board discussion on the system dashboard and the assessment of FIGO staging based on machine learning. The system provides also an alert that signals the degree of discrepancies in the diagnostic results which may impact the discrimination power (**Figure 4**) – the scoring in the dashboard will be low in case of controversial results. From there, the clinical team can navigate through the system, giving priority to the most critical patients (i.e., the patients where the model shows the lowest discrimination power, as in the example in **Figure 5**, where the A.I. model shows a low discrimination power, 66%, due to discrepancies in the different diagnostics);
- as already mentioned, from the single panel view of the critical patients, the MTB can get to a deeper view by analyzing the specific clinical features classification from the three exams (**Figure 1**). This drill-down may highlight clinical features where two exams have led to different interpretations from two specialists (e.g., radiologist and nuclear medicine physician), which in itself would trigger more discussion in the board.

Ultimately, the clinical team may want to compare the eHR items that originated the discrepancies, which would be immediately available in the system (**Figure 2**).

In order to test the effectiveness of the overall approach, the system has been tested with an independent group of 13 patients (whose features have not been used in the Training Set), confirming overall positive performances.

After all the medical reports were retrieved, the NLP system proceeded in classifying all patients in detail through their patterns of clinical features: the predictive model for FIGO staging has shown an accuracy of 93%, substantially confirming the performances observed in the training set.

## Discussion

A proof-of-concept for an integrated framework for automated classification of disease staging, and a Clinical Decision Support System in the multidisciplinary management of LACC is reported.

Using NLP, we have trained and validated a biomedical imaging report analyzer that performs a smart “automated classification” of the LACC stage. As a primary staging method, the algorithm trained using digital MR, PET-CT and EUA reports from the cohort used in the Training Set, achieved excellent accuracy when matched with the prediction of the stage.

The performance compared favorably to clinical staging and was confirmed to the same levels of accuracy when tested in the independent Validation Set. Notably, the reports were performed by different physicians without using a common template, so even though the task for the software was complex and prone to misinterpretation, it was successful.

To the best of our knowledge this is the first prototype aimed at supporting effectively a MTB in the prioritization and analysis of the most critical cases. The intuitive Graphical User Interface allows an easy detection of discrepancies among the imaging reports, so, rather than focusing on the clinical cases without diagnostic uncertainties whose treatment should be an easy skill, the software suggests the specialists to focus their attention on the most critical cases, optimizing both human and time resources, dedicating more quality time to deep discussion and achieving a more robust data-driven consensus.

In a large-volume scenario as well as in a low- resources setting, the implementation of an automatic tool as the one described could have a very relevant impact as supported by the promising results of this proof-of-concept.

Obviously, this tool is not intended to replace the tumor board’s discussion of clinical situations, even in circumstances when the UAE and MR are in agreement. At truth, reports frequently underpin parts that aren’t written, but are assessed in multidisciplinary meetings (e.g. some poor detection of the outer cervical stroma that is not a sure sign of parametrial invasion).It has to be considered as a facilitator of the decisional process and a tool to make MTD meetings go faster even if there are numerous clinical cases to be discussed.

The approach proposed in this paper is – to the best of our knowledge – quite novel and can complement other AI-based technologies experienced in other research projects (4, 14). As an example, radiology-focused applications aim to automate and streamline analytical tasks in order to improve the efficiency, accuracy, and consistency in the interpretation of the radiological imaging (e.g., computer aided detection and diagnosis software, i.e., CADe and CADx). Similarly, computer aided triage (CADt) software analyzes images to prioritize the review of images for patients with potentially time sensitive findings. Another promising area of growth is the use of AI to set up a Clinical Decision Support System for the treatment of cancer (e.g., IBM “Watson for Oncology” software). The latter stores and indexes literature, protocols, and patient charts, learning from test cases; thereafter, all the information input is verified by the experts from Memorial Sloan Kettering Cancer Center (15).

Albeit very focused and specific for LACC, this proof of concept could be easily adapted and extended to other cancer settings, demonstrating the favorable scalability of the provided structure. There is, in fact, much room for re-use of the many pivotal components:

- Extract/Transform/Load (ETL) automated extraction and following NLP clinical features classification;
- machine-learning based predictive model for FIGO staging, which can be trained on different patient set, classification system and endpoints;
- overall navigation and drill-down to different layers of information, to allow the MTB for a data-supported analysis and discussion (thus promoting collaborative methods and integration of skills).
- especially in the Covid 19 era where MTBs are performed increasingly in virtual/online mode, this system offers a remote collaborative platform into the hospital and among hospitals.

Furthermore, as already suggested by Bizzo et al. (16), A.I. can help drive the field toward more structured reporting from different specialists, which is critical for an effective MTB and serves as the basis for a “virtuous cycle” in creating additional data for A.I. to improve upon.

Moreover, a further strength of the proposed approach is represented by the machine-learning and clustering methods - used in connection with NLP and understanding of clinical features from diagnoses – that allowed us to identify patients’ phenotypes which are not characterized only through the FIGO staging and can be especially useful for future prognostic models able to predict the complete pathological response, as well as other prognostic outcomes.

Lastly, new prospective clinical scenarios such as the possibility to introduce into the software other clinical tools that could be useful for early cervical cancer characterization can be speculated. For example, the addition of cervix clinical morphology and characterization by colposcopy images or ultrasound measurements to the MTB Smart Virtual Assistant software could be quite useful in determining whether or not to employ conization in early cervical cancer. Some literature data are already available and could form the basis for a future integration project (17–20).

In terms of future developments starting from this proof of concept, we consider strategic the following key points:

- enlarge the training and validation cohort by recruiting patients coming from our center as well as other institutions: the increased cohort will allow to further improve the NLP effectiveness and predictive system accuracy;
- use of this setting as a base for an end-to-end model; covering also the re-staging and the pathological response definition; in this way, we could be able to provide further insights to the MTB not only at the diagnostics phase, but also along the treatment and the follow-up.
- extend to other languages (e.g., English), possibly integrating existing NLP system for eHR transformation and then connecting our clustering and predictive methodologies: this transformation could allow a widely dissemination.

In conclusion, while this prototype should still be considered as first proof of concept of the possibility of creating a Smart Virtual Assistant for MTB, we believe that this experience discloses a significant benefit in the integration of these automated methods in the collaborative, crucial decisional steps, giving clinicians the opportunity to save time by optimizing the duration of multidisciplinary meetings, to consolidate information and leverage data-driven evidence that would be not achievable in the more traditional settings and decisional workflows.

## Data Availability Statement

The raw data supporting the conclusions of this article will be made available by the authors, without undue reservation.

## Ethics Statement

Ethical approval was not provided for this study on human participants because the project has received approval and has been reviewed by the Scientific Director of IRCCS Policlinico Gemelli. The patients/participants provided their written informed consent to participate in this study. Written informed consent was obtained from the individual(s) for the publication of any potentially identifiable images or data included in this article.

## Author Contributions

Conception and design, GM, SP, and VV. Revision of study design and protocol, GF, GM, AC, RA, LB, and CC. Study coordination, GM, SP, RA, and CM. Acquisition of data and patient recruitment, VP, CC, CI, BG, VR, MG, and VV. Radiotherapy quality check (of protocol), GM, RA, VV, and LB. Data management and statistical analysis, SP, VP, CI, CC, and CM. Revision of adaptation of and final approval of manuscript, all authors. Accountable for all aspects of the work, all authors. All authors contributed to the article and approved the submitted version.

## Conflict of Interest

The authors declare that the research was conducted in the absence of any commercial or financial relationships that could be construed as a potential conflict of interest.

## Publisher’s Note

All claims expressed in this article are solely those of the authors and do not necessarily represent those of their affiliated organizations, or those of the publisher, the editors and the reviewers. Any product that may be evaluated in this article, or claim that may be made by its manufacturer, is not guaranteed or endorsed by the publisher.
